# Frequency Response of Induced-Charge Electrophoretic Metallic Janus Particles

**DOI:** 10.3390/mi11030334

**Published:** 2020-03-24

**Authors:** Chong Shen, Zhiyu Jiang, Lanfang Li, James F. Gilchrist, H. Daniel Ou-Yang

**Affiliations:** 1 Department of Physics, Lehigh University, Bethlehem, PA 18015, USA; chs514@lehigh.edu (C.S.); zhj216@lehigh.edu (Z.J.); lal419@lehigh.edu (L.L.); 2 Emulsion Polymers Institute, Lehigh University, Bethlehem, PA 18015, USA; 3 Department of Chemical & Biomolecular Engineering, Lehigh University, Bethlehem, PA 18015, USA; gilchrist@lehigh.edu; 4 Department of Bioengineering, Lehigh University, Bethlehem, PA 18015, USA

**Keywords:** induced charge electrophoresis (ICEP), Janus particles, optical trapping, phase-sensitive detection, phoretic force spectroscopy, ICEP motility reversal, micro-robotics

## Abstract

The ability to manipulate and control active microparticles is essential for designing microrobots for applications. This paper describes the use of electric and magnetic fields to control the direction and speed of induced-charge electrophoresis (ICEP) driven metallic Janus microrobots. A direct current (DC) magnetic field applied in the direction perpendicular to the electric field maintains the linear movement of particles in a 2D plane. Phoretic force spectroscopy (PFS), a phase-sensitive detection method to detect the motions of phoretic particles, is used to characterize the frequency-dependent phoretic mobility and drag coefficient of the phoretic force. When the electric field is scanned over a frequency range of 1 kHz–1 MHz, the Janus particles exhibit an ICEP direction reversal at a crossover frequency at ~30 kH., Below this crossover frequency, the particle moves in a direction towards the dielectric side of the particle, and above this frequency, the particle moves towards the metallic side. The ICEP phoretic drag coefficient measured by PFS is found to be similar to that of the Stokes drag. Further investigation is required to study microscopic interpretations of the frequency at which ICEP mobility switched signs and the reason why the magnitudes of the forward and reversed modes of ICEP are so different.

## 1. Introduction

Microrobots are considered as a potential future workforce. Some examples of such applications include the use of active Janus particles to enhance optical resolution for measurements of molecular interactions in biological samples [1], enabling the optimization of transportation or navigation [2], self-assembly and formation of microscopic smart materials [3,4], serving as cargo movers for medicine delivery [5], and functioning as micromanipulators or micromixers [6]. Today, robotic devices are made at increasing smaller scales, reaching that of colloidal particles [5]. Among such endeavors are creative efforts to make active colloidal particles that convert energy provided by an external source to kinetic energy in order to move persistently [7,8]. Such active colloids can be powered by a variety of different mechanisms, such as self-thermophoresis [9], chemical decomposition [10], electric fields [11], and magnetic fields [12]. In this work, we apply an AC electric field and use induced-charge-electrophoresis (ICEP) to drive particle motion and address the possibilities of controlling such colloid-based microrobots by their electric frequency response.

**Figure 1:**
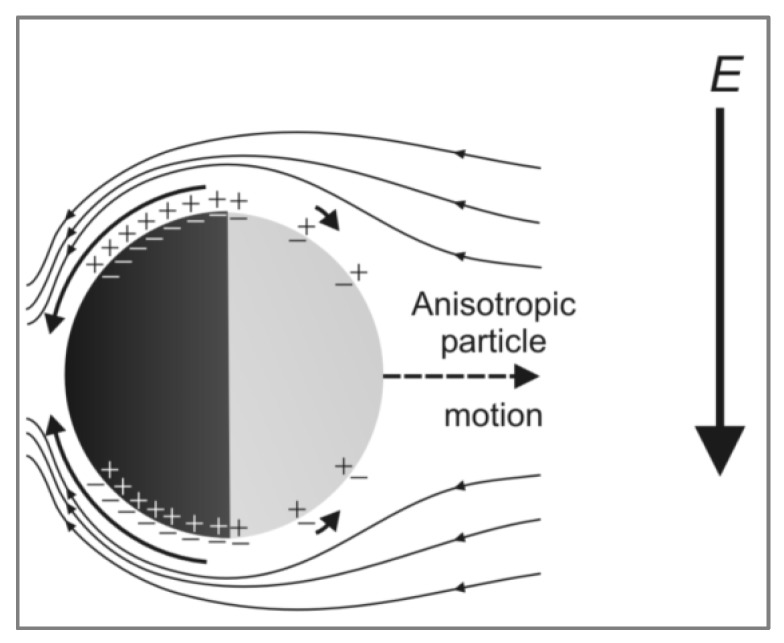
Reproduction of a schematic by Gangwal et al. of a particle in one-half cycle of an AC electric field in the stable configuration. The electric double layer on the gold side (black hemisphere) is more strongly polarized and thus drives a stronger induced-charge-electroosmosis slip (arrows) than the polystyrene side, resulting in induced-charge electrophoresis (ICEP) motion in the direction of the dielectric side. Reprinted with permission [14].

Control of active colloids by external fields has been demonstrated in terms of precise alignment [13], directed control of micro motors [6], and cargo transport [3] for potential microrobot applications. Gangwal et al. [14] used a uniform distributed alternating current (AC) electric field in the kHz frequency range to produce a propulsive metal-dielectric Janus particle via ICEP. A theory for ICEP was first introduced by Squires and Bazant [15]. Based on the theory, an AC electric field could induce different electroosmotic flows around the dielectric surface and around the metallic surface, causing the particle to move. According to the model, the flow around the metallic surface was stronger than the flow around the dielectric surface; the pushing force on the metal surface was higher than the force on the dielectric surface. The net force pushes the particle in the direction from the metal side toward the dielectric side (normal direction), shown in Figure 1.

According to the model by Squires and Bazant [15], the ICEP mobility should decay to zero as the frequency of the AC electric field exceeds the characteristic times for the ions in the electric double layers to respond to the applied electric field. In Mano et al.’s experiments [16], however, a phoretic motion reversal was observed during a frequency scan, i.e., the Janus particle was found to be moving in a direction led by the metallic side. One possible explanation for such a mobility reversal was the reversal of induced-charge electroosmosis (ICEO) flow [15], i.e., the external electric field-induced flows of liquid around the two sides of the particles changed direction [17]. In an alternating current electrophoresis (ACEO) setting [18], the additional nonlinear mechanisms including Faradic reactions [19,20] and steric effects [17] due to ion crowding within the electrical double layers (EDLs), were considered to explain the phenomena. In traveling wave electroosmosis (TWEOF), the motility reversal could be expressed as the electrical body force [21,22]. All these explanations neither can be applied to the Janus particle system [21,22] nor need unreasonable ionic density [17,19,20].

A better understanding of the ICEP motility reversal requires better experiments that can determine the crossover frequency and the phoretic drag coefficient accurately. Using traditional DC measurements to determine the crossover frequency is difficult because the Brownian motion often swamps measurements of the null phoretic motion near the crossover frequency [23]. It is impossible to determine the drag coefficient of any phoretic motion by DC measurements, because the phoretic motion, absent of acceleration, is subject to zero net force, as the drag force perfectly opposes the phoretic force [24].

It is, however, possible to determine the drag coefficient by an AC measurement from the phase delay of the particle’s phoretic motion relative to that of the harmonically varying electrophoretic force, as demonstrated in earlier work [24]. We have used such a detection method to measure the crossover frequency and phoretic force of dielectrophoresis with high precision [23,25]. Briefly, our detection method, phoretic force spectroscopy (PFS), measures the harmonic response of an optically trapped phoretic particle in an amplitude-modulated AC electric field. A lock-in amplifier analyzes the motion of the particle to yield the particle motion’s amplitudes, and phase lags relative to the phase of the harmonic field. The crossover frequency is the frequency at which the phase lag changes by 180 degrees. In this paper, we describe how we use PFS to study the frequency dependence and phoretic drag coefficient of ICEP.

## 2. Materials and Methods

### 2.1. Fabrication of Metal-Dielectric Janus Particles

**Figure 2:**
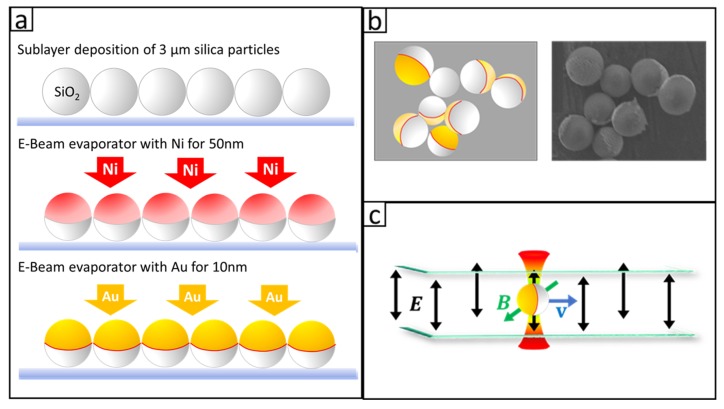
(**a**) Fabrication process of Janus particles. Silica particles were deposited from an aqueous suspension to form a sub-monolayer on a glass substrate, followed by E-beam metal evaporation deposition of Ni and Au. (**b**) On the left is a cartoon showing the shape and geometry of resultant Janus particles, and on the right is a scanning electron microscope (SEM) micrograph of actual particles. (**c**) A depiction of a trapped Janus particle in the sample chamber. The directions of the particle’s motion, the E and B fields, are shown relative to the sample chamber.

We made metallic Janus particles by depositing a thin film of metal on a monolayer of dry silica particles (SS05N, Bangs Laboratories, Inc., Fishers, IN, USA) [26] with a process shown in Figure 2a. A droplet of 3 micron diameter silica particle suspension was deposited on a glass substrate to create a monolayer by a vibration-assisted convective deposition method. The slide covered with a monolayer of particles was loaded into an e-beam evaporator (Indel E-beam Evaporator, International Delta Systems, LLC, Tucson, AZ, USA) to receive first a coating of 50 nm Ni and then a coating of 10 nm Au on the top half of the particle surface. The slide was then placed in a water-filled centrifuge tube and sonicated in a sonication bath (Branson 1510, Branson Ultrasonics Co., Danbury, CT, USA) for 6 h to release the particles into the water for further treatment. Schematics of the Janus particles and a scanning electron microscope (SEM) (JEOL JXA-8900 EPMA Microprobe, JEOL USA, Inc., Pleasanton, CA, USA) micrograph of the Janus particles are shown in Figure 2b. 

### 2.2. Application of AC Electric Field to Drive Janus Particles Based on ICEP

Figure 2c shows the schematic of the sample chamber in which the particle’s motions were examined. A pair of indium-tin-oxide (ITO) coated glass slides, separated by a 50 um spacer of polycarbonate tape (3M 980, 3M, St. Paul, MN, USA), were fixed together by wrapping with the same tape. Copper tapes were attached to the ITO coating on each glass slide to form an electrical contact. An AC electric signal was applied across the pair of ITO slides to create a uniform electric field in the vertical direction. In the presence of the AC electric field, the Janus particles were driven into drift motions in the horizontal direction by ICEP. Since Janus particles settled very close to the bottom of the sample chamber, the particles moved in a two dimensional (2D) plane. 

### 2.3. Application of a Magnetic Field to Fix the Direction of the ICEP Driven Phoretic Motion

While the vertically applied electric field exerted a torque on the Janus particles and aligned the metal cap edge parallel to the E field, the polar axis of the particles still had the freedom to orient freely in the plane perpendicular to the E field. Since the metal cap of the Janus particles exhibited magnetic dipole moment in the polar direction, an external magnetic field was applied to align the Janus particle orientation in the 2D plane perpendicular to the E field. The ICEP force was aligned with the polar direction of the Janus particles, and a magnetic field was used to control the direction of particle movement, as shown by Lin et al. [27] and Han et al. [28] to control and manipulate the metallic Janus particles, respectively. In this work, the magnetic field was generated by four 15 mm × 6.5 mm × 3 mm rectangular commercial Neodymium magnets (rectangular magnets, Theodora LLC, Seattle, WA, USA). The four magnets were divided into two groups, with two magnets in each group. Two groups of magnets were placed 10 cm apart and with the S and N poles facing each other in the horizontal plane. The sample chamber was placed at the center of the two groups of magnets. The intensity of the magnetic field at the location of the sample chamber was measured by Gauss meter (SJ200, Guilin Senjie Technology Co., Ltd., Guilin, China) to be 1.0±0.1 mT.

### 2.4. Position Detection of Phoretic Particles in 2D Using Image Analysis

**Figure 3:**
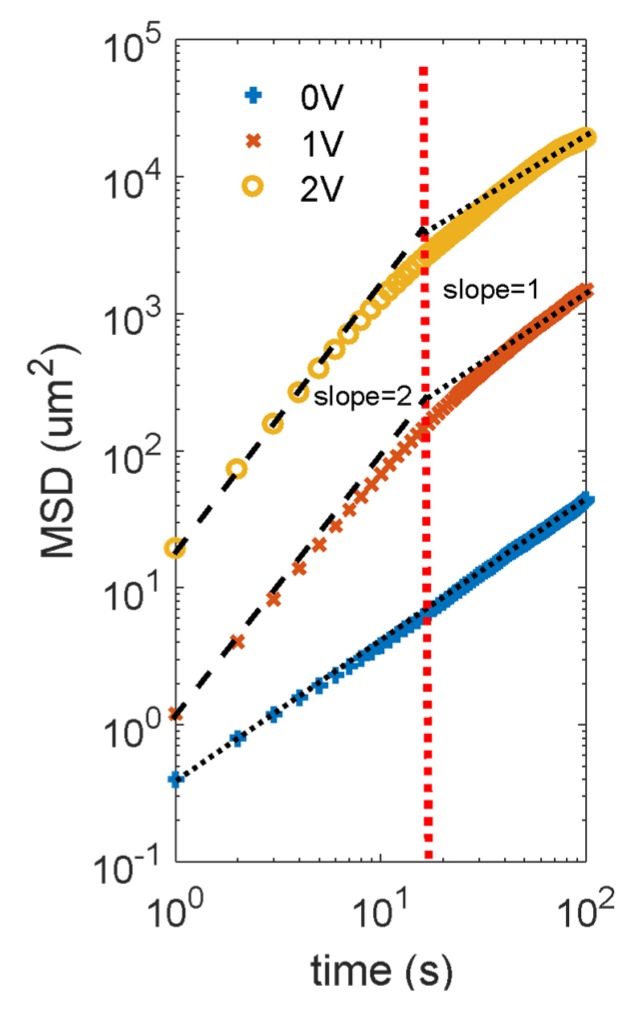
Mean-squared-displacement (MSD) of ICEP-driven metallic Janus particle under 5 kHz AC electric field. The extrapolated dash lines are from ballistic motion (the slope of 2) to the active diffusive motion (the slope of 1). The red vertical dotted line represents the particle’s rotational relaxation time.

The particle’s motion was measured by video imaging using a 20× objective lens on an inverted microscope (Olympus IX-81, Olympus Corporation, Tokyo, Japan). The videos of the particle movement were recorded by a charge-coupled device (CCD) camera (Sony Sscm256, Sony Corp., Tokyo, Japan) at a frame rate of 1 frame per second for a typical duration of a few minutes. The video recordings were analyzed using a particle tracking program (MOSAIC Suit on ImageJ, Max Planck Institute of Molecular Cell Biology and Genetics (MPI-CBG), Dresden, Germany.) [29] to extract the trajectories of particles in the experimental chamber. Mean-square-displacement (MSD), defined as $<\left|x{\left(t\right)}^{2}-x{\left(t+t_{0}\right)}^{2}\right|>$, was calculated by taking the average of the MSD for all particles in the field of view. The experimental MSD vs. time for 3 µm metallic Janus particles at varying applied voltage over a fixed gap distance (50 µm) between the electrodes is shown in Figure 3.

### 2.5. Trapping and Manipulation of a Janus Particle

**Figure 4:**
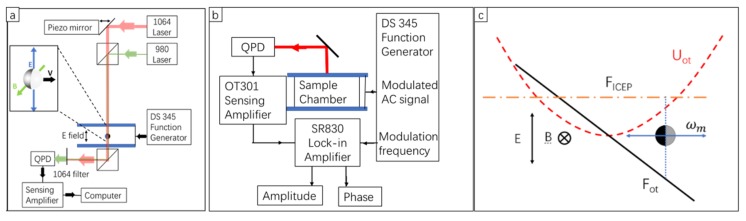
Phoretic force spectroscopy (PFS) set-up and force diagram. (**a**) Lightpath diagram of PFS. A 1064 nm laser was used to trap a Janus particle. A 980 nm tracking laser was co-aligned with a 1064 nm trapping laser. The 980 nm tracking laser was detected by a quadrant photodiode (QPD). A magnet provided an in-plane magnetic field to further align the Janus Particles (insert). (**b**) Signal chain diagram. A lock-in amplifier analyzed the signal of particle position with the modulation signal as a reference. (**c**) Force diagram of an optically trapped Janus particle under a modulated AC electric field. U_ot_ is the potential of a particle in the trap. F_ICEP_ (active force) and F_ot_ (trap force) are in balance at a non-center spot. Electric field (E) is in the z-direction, and the magnetic field is in the y-direction. The orientation of the Janus particle is in the x-direction. The particle oscillates, and the oscillation frequency is the same as the frequency of the modulation signal.

A 1064 nm wavelength laser (Nd:YVO_4_ 1064nm diode-pumped solid-state CW laser, Spectra-Physics, Santa Clara, CA, USA) was used to create an optical trap, as shown in Figure 4a. The trap confines the Janus particle in a quadratic potential well [30]. The displacement of the particle in a trap, too small for video analysis to pick up, was tracked by a tracking beam (980 nm CL-2000 diode-pumped crystal laser, CrystaLaser, Reno, NV, USA) aligned collinearly with the 1064 nm laser. The tracking beam was received by a quadrant photodiode (QPD) that measured the position of the particle to give 5 nm spatial resolution at 1 kHz sampling rate. We used this setup to measure coefficients of the Stokes’ drag [31] An AC voltage was applied using a function generator (Stanford Research DS345, Stanford Research Systems, Sunnyvale, CS, USA) across the electrodes in the experimental chamber. The electric field produced by the electric potential across the electrodes drives ICEP and makes the particles an active Brownian particle. Active driving, trapping, and tracking of a metal-dielectric Janus particle was achieved in the same setup. A magnetic field was added to control the ICEP motion, so the particle moved linearly. 

### 2.6. Phoretic Force Spectroscopy

Phoretic force spectroscopy (PFS) was used to measure the phoretic force of the Janus particles in the electric field [23]. Over a range of frequencies of the ICEP driving field, the motion of a particle in an optical trap was analyzed with an amplitude-modulated AC electric field. By using a lock-in amplifier, we measured the amplitudes and phase lags of an oscillated particle with the modulation signal as a reference. Not only the amplitude of the motion but also the phase relative to the driving force were obtained. The directional change of ICEP was detected when a 180-degree phase change was observed.

The lock-in amplifier (Stanford Research SR830 DSP Dual Phase Lock-In Amplifier, Stanford ResearchSystems, Sunnyvale, CA, USA) required a signal input and a reference input, as shown in Figure 4b. The reference was from the modulation frequency output of the function generator (Stanford Research, DS345, Stanford Research Systems, Sunnyvale, CA, USA). The signal was from the quadrant photodiode (homemade QPD) which measured the position of the particle. Here, the signal was amplified by a sensing amplifier (On-Trak OT301, On-Trak Photonics, Irvine, CA, USA) before it went into the lock-in amplifier. The amplitude (*A*) and the phase lag (*δ*) of the particle motion were measured by the lock-in amplifier.

The motion of the particle was analyzed as follows. Janus particles are assumed to have a constant speed, v, in an AC electric field with amplitude E. The speed is proportional to the square of the AC electric field strength, $v=\beta E^{2}$, where β is ICEP mobility. In the water, the drag force equals the ICEP forces, $F_{e}=\zeta_{ICEP}v=\zeta_{ICEP}\beta E^{2}$, where $\zeta_{ICEP}$ is the drag coefficient. At any instance, tweezer force, drag force, and resistance are in balance, so the Langevin equation of the position, x, of the particle is:

(1) $$\frac{dx}{dt}+\frac{k_{ot}x}{\zeta_{ICEP}}=\beta E^{2}$$

where $k_{ot}$ is the spring constant of the optical trap. In our experiment, the electric field with 30% depth modulation was $E=E_{0}\left[0.85+0.15\cos\left(\Omega_{M}t\right)\right]\cos\left(\Omega_{B}t\right)$, where the $\Omega_{M}$ is amplitude modulation frequency. Equation (1) can be rewritten to be

(2) $$\begin{array}{c} \frac{dx}{dt}+\frac{k_{ot}x}{\zeta_{ICEP}}=\beta E_{0}^{2}{\left[0.85+0.15\cos\left(\Omega_{M}t\right)\right]}^{2}\cos{\left(\Omega_{B}t\right)}^{2}\\ =\beta E_{0}^{2}\left\{0.832+0.255\cos\left(\Omega_{M}t\right)+0.01125\cos\left(2\Omega_{M}t\right)\right\}\left(\frac{1}{2}+\frac{1}{2}\cos\left(2\Omega_{B}t\right)\right)\\ =\beta E_{0}^{2}\{0.416+0.1775\cos\left(\Omega_{M}t\right)+0.005625\cos\left(2\Omega_{M}t\right)+0.416\cos\left(2\Omega_{B}t\right)+\\ \frac{1}{2}[0.1775\cos\left((2\Omega_{B}-\Omega_{M})t\right)+0.1775\cos\left((2\Omega_{B}+\Omega_{M})t\right)]+\frac{1}{2}[0.005625\cos\left(2(\Omega_{B}-\Omega_{M})t\right)+\\ 0.005625\cos\left(2(\Omega_{B}+\Omega_{M})t\right)]\} \end{array}$$

The amplitudes of the high-frequency terms (higher than $\Omega_{B}$) are small due to water damping [32]. Thus, we can ignore the higher frequency term and only consider the first three terms (in boldface in Equation (2)). The steady-state solution for Equation (2), x(t) has a term of a DC offset, a first harmonic term and a second harmonic term, each with amplitude $A\left(\Omega_{M}\right)$, and $A\prime \left(2\Omega_{M}\right)$ and phase delay $\delta \left(\Omega_{M}\right)$ and $\delta \prime \left(2\Omega_{M}\right)$, respectively, i.e.,

(3) $$x\left(t\right)=x_{DC\;offset}+A\left(\Omega_{M}\right)e^{i\left(\Omega_{M}t-\delta \right)}+A^{\prime }\left(2\Omega_{M}\right)e^{i\left(2\Omega_{M}t-\delta^{\prime }\right)}$$

The frequency-dependent amplitudes *A*, *A′* and the phase delays δ, δ′ are

(4) $$A\left(\Omega \right)=\frac{0.255\beta E_{0}^{2}\zeta_{ICEP}}{\sqrt{k_{ot}^{2}+{\left(\zeta_{ICEP}\Omega_{M}\right)}^{2}}}$$

(5) $$A^{\prime \left(2\Omega \right)}=\frac{0.01125\beta E_{0}^{2}\zeta_{ICEP}}{\sqrt{k_{ot}^{2}+{\left(2\zeta_{ICEP}\Omega_{M}\right)}^{2}}}$$

(6) $$\delta \left(\Omega \right)=\tan^{-1}\frac{\zeta_{ICEP}\Omega_{M}}{k_{ot}}$$

(7) $$\delta^{\prime \left(2\Omega \right)}=\tan^{-1}\frac{2\zeta_{ICEP}\Omega_{M}}{k_{ot}}$$

Since the second harmonic ($2\Omega_{M}$) terms have a much smaller amplitude than that of the fundamental frequency terms we use the $\Omega_{M}$ term for further calculation. The two parameters of interest of this study, the mobility β and the phoretic coefficient $\zeta_{ICEP}$, can be determined in terms of the experimental measurables, $A\left(\Omega_{M}\right)$, $\delta \left(\Omega_{M}\right)$ and $k_{ot}$. According to Equations (4) and (6) above, we have the following relationships:

(8) $$\beta =\frac{A\left(\Omega_{M}\right)\Omega_{M}}{0.255E_{0}^{2}sin\delta \left(\Omega_{M}\right)}$$

(9) $$\zeta_{ICEP}=\frac{k_{ot}}{\Omega_{M}}tan\delta \left(\Omega_{M}\right)$$

Although the $A\left(\Omega_{M}\right)$ and $\delta \left(\Omega_{M}\right)$ are a function of modulation frequency, according to our model, the β and $\zeta_{ICEP}$ should be independent of the modulation frequency. The β and $\zeta_{ICEP}$ were tested to be the same with modulation frequency from 1 Hz to 3 Hz. However, the $A\left(\Omega_{M}\right)$ and $\delta \left(\Omega_{M}\right)$ decreased to about zero when $\Omega_{M}$ was larger than 3 Hz. Thus, we set the modulation frequency $\Omega_{M}$ to be 1 Hz in our experiment for the best signal and use a lock-in amplifier to measure $A\left(\Omega_{M}\right)$ and $\delta \left(\Omega_{M}\right)$ from which we calculated the mobility β and the phoretic coefficient $\zeta_{ICEP}$ of ICEP-driven metallic Janus particles 

The force diagram of a metallic Janus particle under the modulated AC electric field was shown in Figure 4b. The optical force, $F_{ot}$, was generated from the optical trap. The ICEP force was generated from the AC electric field. Since the electric field and magnetic field were applied to fix the orientation of the Janus particle, the direction of the ICEP force was fixed. The average ICEP force was non-zero due to the 30% amplitude modulation. The particle oscillated around an offset that was predicted by Equation (3). The 30% amplitude modulation generated an offset which became the balance point between the force from the trap and the force from ICEP.

We conducted an independent experiment (passive microrheology [33]) to measure the trap stiffness $k_{ot}$ using the same optically trapped particle without the presence of the AC electric field. According to the equal partition relationship, we have

(10) $$\frac{1}{2}k_{ot}x^{2}=\frac{1}{2}k_{B}T$$

where $\langle x^{2}\rangle$ is the mean-squared displacement of the particle in the trap, $k_{B}$ the Boltzmann constant, and T the absolute temperature. We determined $k_{ot}$ by measuring the mean-squared displacement $\langle x^{2}\rangle$.

### 2.7. Stokes’ Drag Coefficient of the Particle near the Bottom of the ITO Glass Chamber

We used the active microrheology to determined the Stokes’ drag coefficient, $\zeta_{Stokes}$ [31]. We applied an oscillatory optical force to a trapped bead and analyzed the oscillatory motion of the particle to determine the Stokes’ drag coefficient of the particle in 2D near the bottom of the glass chamber. The trapped bead was forced to oscillate along the x-direction by the oscillating tweezers driven by the piezoelectric lead zirconate titanate (PZT)-controlled mirror. The equation of motion of the trapped bead can be written as

(11) $$\zeta_{Stokes}\frac{dx}{dt}=k_{ot}\left[A_{0}\cos\left(\omega t\right)-x\right]$$

where $A_{0}$ is the maximum displacement of the trap and ω is the angular velocity of the oscillation. The steady-state solution of the motion is:

(12) x=D(ω)cos(ωt+δ″)

where D(ω) is the amplitude of displacement and δ″ is the phase lag. Both values were from the lock-in amplifier. The frequency-dependent amplitudes *D* and the phase delays δ″ are

(13) $$D\left(\omega \right)=\frac{A_{0}k_{ot}}{\sqrt{k_{ot}^{2}+{\left(\zeta_{Stokes}\Omega \right)}^{2}}}$$

(14) $$\delta \prime \prime \left(\omega \right)=\tan^{-1}\frac{\zeta_{Stokes}\Omega }{k_{ot}}$$

Thus,

(15) $$\frac{\zeta_{Stokes}\omega }{k_{ot}}=tan\delta \prime$$

Since we already know $k_{ot}$ from above, we can determine the Stokes drag coefficient $\zeta_{Stokes}$ of the Janus particle near the bottom of the glass plate.

We estimate the distance between the particle bottom and the ITO surface with the Faxen equation. Faxen law describes the drag coefficient change near a flat surface due to the non-slipping boundary condition, which can be written as [34]

(16) $$\frac{\zeta_{Stokes}}{6\pi \eta r}=\frac{1}{1-\frac{9}{16}\frac{r}{h}+\frac{1}{8}{\left(\frac{r}{h}\right)}^{3}-\frac{45}{256}{\left(\frac{r}{h}\right)}^{4}-\frac{1}{16}{\left(\frac{r}{h}\right)}^{5}}$$

where η is the viscosity of water, *r* is the radius of Janus particle and *h* is the distance between the center of particle and the surface. Since we already know $\zeta_{Stokes}$, we can determine the gap (*h* − *r*) between the particle bottom and the ITO surface.

## 3. Results and Discussions

### 3.1. ICEP Movement of an Unconfined Janus Particle in 2D

Due to the high-mass density and micron sizes, these Janus particles tended to sediment to the bottom of the sample chamber. These particles did not stick to the substrate presumably due to Coulomb repulsion between the same negatively charged surface of the indium–tin–oxide (ITO) coated glass slide and that of the Janus particle surfaces. The electric repulsion was sufficiently strong to elevate the particles at a small distance above the bottom surface. We estimated the electrical repulsive force using Derjaguin–Landau–Verwey–Overbeek (DLVO) theory [35] and Derjaguin and superposition approximations [36],

(17) $$F=4\pi \varepsilon \kappa r\phi_{Janus}^{eff}\phi_{ITO}^{eff}exp\left(\kappa z\right)$$

where ε is the permittivity of water, κ is one over the Debye screening length, *r* is the radius of Janus particle, $\phi_{Janus}^{eff}\;and\;\phi_{ITO}^{eff}$ are the effective potential of the Janus particle and ITO surface, respectively; z is the distance between the bottom surface of the Janus particle and the ITO surface, and

(18) $$\phi_{\mathrm{Janus},\;\mathrm{ITO}}^{eff}=\left(\frac{4k_{B}T}{e}\right)\tan h\left(\frac{e\phi_{Janus,ITO}}{4k_{B}T}\right)$$

We measured the zeta potential of the Janus particle ($\phi_{Janus})$ to be −17.01±1.13 mV (ZetaPlus Brookhaven instrument). We used a published value for the zeta potential of ITO surface ($\phi_{ITO})$ in DI water to be −32.7±0.2 mW [37] to calculate effective potential [36]. We used the conductance of DI water 167 ms (ZetaPlus, Brookhaven Instruments Corp., Holtsville, NY, USA) to determine the Debye screening length to be 5.55 nm. The permittivity of water was $80.2\varepsilon_{0}$ at 25 °C, where $\varepsilon_{0}$ is the permittivity of vacuum [38]. The gravitational force of 3 μm Janus particle was 0.23 pN, calculated with ∆ρVg, where ∆ρ is the mass density difference of silica ($2.65\;g/{\mathrm{cm}}^{3}$) [39] and water ($1.00\;g/{\mathrm{cm}}^{3}$), V was the volume of particle, and g was gravity acceleration. Thus, the gap distance between the particle’s bottom surface and the ITO surface was 47±8 nm, which was the balance point of the particle at which the electric repulsive force and gravitational force were equal to each other.

In the absence of an electric field, Janus particles moved as Brownian particles in a 2-dimensional (2D) plane defined by the glass substrate. Video analysis of the particles’ motion in 2D yielded a 2D mean-squared-displacement (MSD) shown in Figure 3. The MSD curve in the absence of the applied electric field was characteristic of a Brownian particle. Using $D_{t}=\frac{d\left(MSD\right)}{4dt}$ and the measured MSD curve, we determined the diffusivity to be $0.053\pm 0.008\;\mu m^{2}/s$ in the absence of the applied electric field. With this diffusivity, we calculated the drag coefficient using the Stokes-Einstein relation [40]. The drag coefficient was determined to be $7.86\pm 1.37\times 10^{-8}\;\mathrm{Ns}/m$. Using Faxen’s law [34], we determined the distance between the particle’s bottom surface and the ITO surface to be 12.8±0.2 nm; the gap size was about 4 times smaller compared to the distance estimated by the balance between the electrostatic repulsion and the weight of the particle. The discrepancy was probably caused by the overestimation of the zeta potentials of the particle surface, and the ITO surface since during the expriment, the pH value of the water could be ~5.6 instead of 7.0, which would reduce the thickness of the EDL of both surfaces. 

When an AC electric field was applied, the Janus particles underwent induced-charge dielectrophoresis (ICEP). Modeling the ICEP driven particle as an active swimmer, its MSD vs. time curve was predicted by Marchetti [41] as Equation (19).

(19) $$MSD=4D_{t}t+2v_{0}^{2}\tau_{r}\left[t-\tau_{r}\left(1-e^{-\frac{t}{\tau_{r}}}\right)\right]$$

According to the equation, a log-log plot of the MSD vs. time curve would yield the active ICEP speed from the intercept of the portion where the slope is 2. Equation (19) could be deducted to $MSD=v_{0}^{2}t^{2}$, during the time interval between $\tau_{r}$ and $v_{o}^{2}/4D_{t}$. This deduction works when the $v_{o}^{2}/4D_{t}$ was larger than $\tau_{r}$. The vertical axis intercept of the ballistic motion section of the curve was $2ln\left(v_{0}\right)$, and $v_{0}$ was calculated from the experimental intercept value. 

**Figure 5:**
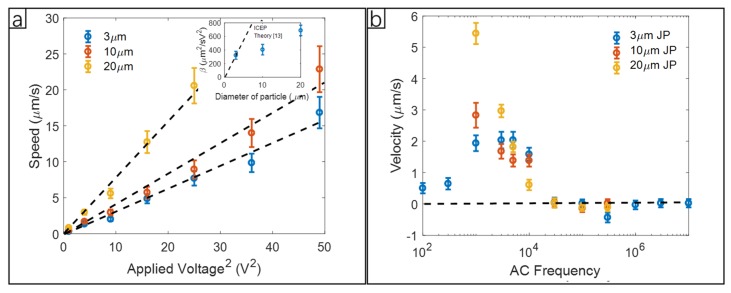
ICEP of a Janus particle without confinement. (**a**) ICEP speed of 3 μm, 10 μm, and 20 μm Janus particles under different applied voltage at 5 kHz. Insert: β vs. the diameter of the particle. The dashed line is the prediction using Equation (20). (**b**) The velocity of 3 μm, 10 μm, and 20 μm diameter particles under the potential difference 2 V over a 50 μm gap between the electrodes and with different frequencies from 100 Hz to 1 MHz. The positive velocity (normal direction) is defined as moving toward the dielectric side.

We examined the size-dependent speed of the Janus particles under the same experimental conditions. The relationship between ICEP active velocity and applied voltage at 5 kHz was shown in Figure 5a. The speed of 3 μm, 10 μm, and 20 μm of metallic Janus particles were nearly proportional to the square of applied voltage as predicted theoretically by Squires and Bazant [15] for normal ICEP at low frequency. The metallic Janus particle driven by ICEP with movement towards the dielectric side had a speed of:

(20) $$v\left(ICEP\right)=\frac{9}{64}\frac{\epsilon a}{\eta \left(1+\delta \right)}E^{2}$$

where ϵ is the electric permittivity, η is the viscosity of the bulk solvent, a is the radius of the particle, *E* is the electric field, and *δ* is the ratio between the capacitances of the compact and diffuse layer in the electric double layer. With a uniform thickness in the experimental cell, the ICEP velocity should be proportional to the square of the voltage according to this theory, which agreed with Figure 5a, as well as Gangwal et al.’s experiment data [14]. The size dependence, which was predicted to be linear with particle diameter, however, was not in agreement with our experimental results. In this work, measured *v*(ICEP) ratios of 3:3.9:6.6, shown in the Figure 5a insert, were lower than the theoretical prediction, which was 3:10:20. It is possible that the discrepancy in size dependence for the largest particles of the group might be due to increased hydrodynamic drag between the bottom of the particle and the glass substrate since gap sizes decrease as the particle sizes increase in our experiment. It is also possible that the discrepancies might be caused by the fact that the parameters δ in these three cases were different.

The frequency-dependent ICEP velocity was measured and shown in Figure 5b. There was a characteristic frequency $f_{f}$ at which the ICEP velocity was at the maximum in the normal direction (from the metal side toward the dielectric side). As the frequency increases, we observed that the velocity first decreases gradually to zero, then the direction of motion reverses at a frequency $f_{c}$, the crossover frequency. The negative velocity (the reverse direction) increased as the frequency continuously increased until a peak was reached at a frequency $f_{r}$, after which the ICEP velocity reaches zero at a very high frequency. 

According to Squires et al. [15], the ICEP movement is generated by slip flow around the particle which appears in a certain range of the driving frequency, $\omega_{e}\leq \omega \leq \omega_{p}$. The lower bound $\omega_{e}$ is the minimium charging frequency of the electrode. Here, $\omega_{e}=\frac{D}{\lambda L}$ and *D* is the ion diffusion coefficient, *λ* is the Debye length, and *2L* is the electrode separation. The upper bound, $\omega_{p}$ is the maximum formation frequency of induced ionic cloud screening on the particle, with $\omega_{p}=\frac{D}{\lambda R}$, and *R* is the particle radius. For our experiment the $\omega_{e}=1\;\mathrm{kHz}$, and for the 3 μm particle, $\omega_{p}=30\;\mathrm{kHz}$. In Figure 5b, our experiment shows there was a peak for 3 μm particles in velocity between 1 kHz–5 kHz. The larger 10 μm and 20 μm particles archived maximum speed at even lower frequencies. The frequency range predicted by Squires does include these frequencies where maximum speeds occurred in our experiments. When testing the frequency response of 10 μm and 20 μm diameter particles at frequencies below 1 kHz, Joule heating was found to not be negligible; water vapor bubbles were observed to form at these low frequencies. For frequencies in the range of 100 kHz to 1 MHz, reversed ICEP was observed for the 3 μm particle, where the particle moved towards the metal side. The reversed ICEP had a much lower speed at the same applied voltage (2 V). The 3 μm Janus particle seemed to have a higher reversed ICEP speed in comparison to 10 μm and 20 μm particles. The crossover frequencies for these particles were difficult to determine by video imaging methods because video analysis was not sensitive enough for particles moving at low speeds and when displacements were small.

### 3.2. ICEP Movements in 1D of a Phoretic Particle Confined in a Quadratic Potential

The use of PFS to detect the phoretic motion requires the orientation of the Janus particle to be fixed by a magnetic field, and the amplitude of the AC electric field be modulated. Two permanent magnets applied the magnetic field in a direction perpendicular to the vertical AC electric field. Janus particles with a ferromagnetic Ni layer in the metal cap were used to align the direction of the particle’s largest magnetic moment to the B field. The magnetic field fixed the direction of one axis, and the AC electric field fixed the direction of the other axis of the magnetic Janus particle, thus maintaining the orientation of particles in-plane. With the application of the AC electric field and the DC magnetic fields, the orientation of the particle was fixed in a direction perpendicular to both fields.

We used PFS to determine the crossover frequency and phoretic drag coefficient of the phoretic Janus particle which moved in a linear motion. According to Equation (3), particle motion had two harmonic components and an offset. The lock-in amplifier calculated the amplitude and the phase lag of the particle motion with a frequency component in the first harmonic of the amplitude modulation (AM) frequency. Using PFS, the crossover frequency was measured at the point when the phase lag signal shifted 180 degrees. Using Equations (8) and (9), we calculated the drag coefficient and mobility of the ICEP-driven motion under a range of base wave frequencies (1 kHz–1 MHz).

**Figure 6:**
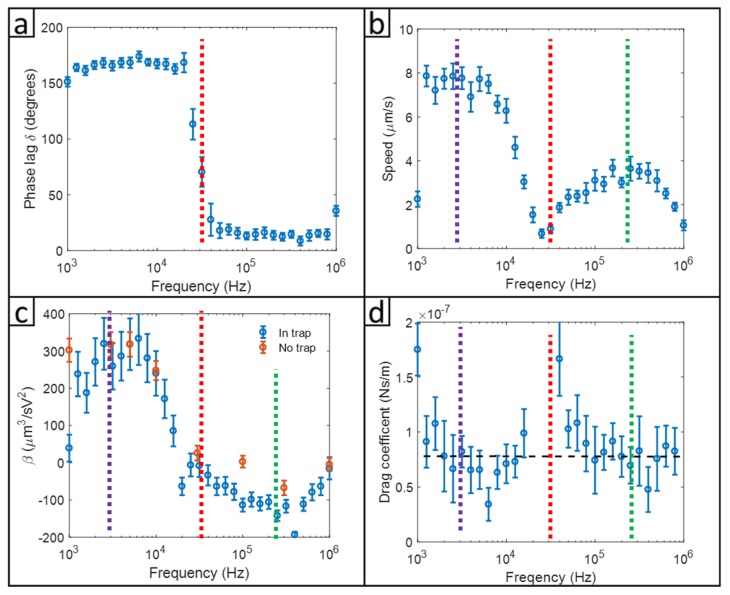
Experimental results of PFS at 0.1 V/μm of the carrier frequency of the electric field from 1 kHz to 1 MHz with 1 Hz amplitude modulation frequency at 30% modulation amplitude. The vertical dash rad lines $\left(f_{c}\right)$ represent the crossover frequency at 27 kHz. The vertical dash green line $\left(f_{r}\right)$ represents the maximum reversal velocity at 220 kHz. The vertical dash purple line $\left(f_{f}\right)$ represents the maximum forward velocity at 1–5 kHz. (**a**) Phase delays of a 3 μm Janus particle. (**b**) Speeds of the same 3 μm Janus particle. (**c**) Comparison of the Beta values of the Janus particles measured with trapping (Equation (3)) and without trapping. (**d**) Drag coefficients determined from the phase delay data.

Figure 6a shows a plot of the phase shift vs. frequency. A 180-degree phase shift between 20 kHz to 30 kHz indicated the normal direction of motion changed into the reverse direction; the frequency at which the 180-degree phase shift was identified as the crossover frequency. Figure 6b shows the speed of the Janus particle as calculated by $v=\Omega_{M}A\left(\Omega_{M}\right)$, i.e., the maximum speed during the oscillating movement under amplitude-modulated ICEP. At frequencies lower than the crossover frequency (~27 kHz), the particle moved towards the dielectric side, defined as the positive velocity (normal direction). At frequencies higher than the crossover frequency of 27 kHz, the particle moved with the metal side in the front. It is informative to compare the crossover frequency of the 3 μm Janus particle with other experiments. Our crossover frequency for the 3 μm Janus particle was 30 kHz. Mano et al.’s experiment on a 3 μm rotational Janus particle showed a cross-over frequency at 22 kHz [16]. Suzuki et al.’s work, also using a 3 μm Janus particle, showed a cross-over frequency at around 30 kHz [42]. The reversal of ICEO often showed smaller crossover frequencies of around 5 kHz [17]. The frequency scales, in the range 5 kHz to 30 kHz, revealed a length scale of 0.2~0.6 μm using the length scale defined by $L=\sqrt{D_{ion}/f_{c}}$. The ionic diffusivity, $D_{ion}$, of K^+^ or Cl^−^ is about $2\times 10^{-9}{\;m}^{2}/s$ [39]. The 30-microsecond time scale revealed by the crossover frequency was on the same order of magnitude of the diffusion time for ions to diffuse the perimeter of the Janus particle, suggesting that the relaxation of the electrokinetic flows in the electric double layer must play a significant role.

Frequency-dependent ICEP mobility β calculated according to Equations (8) and (9), is shown in Figure 6c. The frequency when the speed approached zero in Figure 6c is in agreement with the frequency at which the phase delay changed by 180 degrees. The frequency at which the reversed ICEP speed occurred was the same as that observed in the frequency-dependent β or the frequency-dependent speed. The β vs. frequency curve looked different from that of the speed vs. frequency because of the sinδ term in the denominator of the right side of Equation (8). Using amplitude-modulated ICEP, the oscillation of the driving force had a phase lag (δ) relative to the particle motion. The in-phase term of the driving force was $0.1275E_{0}^{2}sin\delta$, which was used to calculate the β. The out-off-phase term of the driving force was $0.1275E_{0}^{2}cos\delta$, which was used to balance the optical trap force. Mobility β obtained by the AM modulated, and the phoretic force spectroscopy agreed with the value of the 2D measurements for particles not confined by the optical trapping. The maximum speed in the normal direction was at a frequency of 1 kHz–5 kHz, and the peak frequency for the reverse direction was around 200 kHz. We don’t know exactly why the reversed ICEP had much slower mobility than that of the forward motion. A few papers [14,15,16,17,18,19,20] that mentioned the frequency-dependent reversal of electroosmotic flows did not propose mechanisms that would explain the reversal.

We determined the phoretic drag coefficient ξ according to Equation (9), shown in Figure 6d. The drag coefficients were found to be independent of frequency except a few outliers obtained when the particle had very low moving speeds (near crossover frequency) with large error bars. Taking the average over all the frequency, we determined the ICEP drag to be $7.5\pm 2.7\times 10^{-8}\;N\cdot s/m$. The Stokes drag coefficient, measured by oscillating tweezers [29,41] for the same Janus particle in the absence of ICEP, was $7.9\pm 1.4\times 10^{-8}\;N\cdot s/m$, similar to the ICEP drag coefficient.

## 4. Conclusions

This paper reports a study that used phoretic force spectroscopy (PFS) to determine the crossover frequency, the maximum phoretic speeds in the normal and the reverse direction, ICEP mobility, and the ICEP drag coefficient. The direction of the ICEP-driven metallic-dielectric Janus particles was in the normal direction at low frequencies and the reversed direction at high frequencies. Phoretic mobility was proportional to the square of an applied electric field. Our data are in agreement with prior experimental studies. However, our PFS detection method provided better accuracy in determining the crossover frequencies.

The 30-microsecond time scale revealed by the crossover frequency is on the same order of magnitude as the diffusion time for ions to diffuse the perimeter of the Janus particle, suggesting the relaxation of the electrokinetic flows in the electric double layer must play a role. This study found the ICEP drag coefficients of the forward and the reversed motion were similar, and both are comparable to the Stokes drag. Unfortunately, large error bars in our data did not permit more detailed analysis to distinguish the ICEP drag from the Stokes drag. Microscopic interpretations of both the frequency at which ICEP mobility switched signs could benefit from the accurately determined crossover frequencies. The reason for the large difference between the magnitudes of ICEP mobility in the forward and reversed modes of ICEP requires further study.

With the demonstrated ability to determine the high-resolution frequency-dependent response function of the metallic Janus particle by a phase-sensitive detection method, this study can help broaden the application areas and enhance the performance of current applications of colloidal microrobots. For example, a more accurately determined crossover frequency can provide more precise sorting or separation of particles by exposing unsorted particles to judiciously controlled frequencies in a microfluidic channel setting. Metallic Janus particles coated with layers of different magnetic properties exposed in cleverly arranged magnetic fields could be used to transport different kinds of medicines to different locations. Combining the use of electric fields with different frequencies and/or an added magnetic field might also be useful to control the self-assembly of active colloids to achieve complex and time-varying microstructures.
